# Supplementary material for: Asynchronous magnetic resonance elastography: Shear wave speed reconstruction using noise correlation of incoherent waves
Source: Magn Reson Med. 2022 Oct 27;89(3):990–1001. doi: 10.1002/mrm.29502 (PMC9792433; doi:10.1002/mrm.29502)
Supplement: Supplementary file 1 — DATA S1 MATLAB code used to generate the numerical simulations of Figure 1 [file MRM-89-990-s002.zip › k-Wave/helpfiles/filterTimeSeries.html]

filterTimeSeries :: Functions (k-Wave)


# filterTimeSeries

Filter signal using the Kaiser windowing method.

## Syntax

```
filtered_signal = filterTimeSeries(kgrid, medium, signal)
filtered_signal = filterTimeSeries(kgrid, medium, signal, ...)
```

## Description

`filterTimeSeries` filters an input time domain signal using a low pass filter applied by `applyFilter` with a specified cut-off frequency, stop-band attenuation, and transition bandwidth. It uses the Kaiser Windowing method to design the FIR filter, which can be implemented as either a zero phase or linear phase filter. The cutoff frequency is defined by a minimum number of points per wavelength. A smoothing ramp can also be applied to the beginning of the signal to reduce high frequency transients.

## Inputs

|  |  |
| --- | --- |
| `kgrid` | k-Wave grid object returned by kWaveGrid |
| `medium` | k-Wave medium structure |
| `signal` | the time domain signal to filter |

## Optional Inputs

Optional 'string', value pairs that may be used to modify the default computational settings.

| Input | Valid Settings | Default | Description |
| --- | --- | --- | --- |
| `'PlotSignals'` | *(Boolean scalar)* | `false` | Boolean controlling whether the time signal is displayed before and after filtering. |
| `'PlotSpectrums'` | *(Boolean scalar)* | `false` | Boolean controlling whether the amplitude spectrum is displayed before and after filtering. |
| `'PPW'` | *(integer numeric scalar)* | `3` | The number of points per wavelength used to compute the filter cutoff frequency (setting to 0 turns off the filtering). |
| `'RampPPW'` | *(integer numeric scalar)* | `0` | The number of points per wavelength used to compute the length of the cosine start-up ramp (setting to 0 turns off the start-up ramp). |
| `'StopBandAtten'` | *(numeric scalar)* | `60` | Attenuation in decibels in the filter stop band. |
| `'TransitionWidth'` | *(numeric scalar)* | `0.1` | Size of the transition relative to the temporal sampling frequency. |
| `'ZeroPhase'` | *(Boolean scalar)* | `false` | Boolean controlling whether a causal or zero phase filter is applied. |

## Outputs

|  |  |
| --- | --- |
| `filtered_signal` | the filtered time signal |

## Examples

- Monopole Point Source In A Homogeneous Propagation Medium
- Dipole Point Source In A Homogeneous Propagation Medium
- Simulating Transducer Field Patterns
- The Doppler Effect
- Diffraction Through A Slit
- Simulations In Three-Dimensions
- Focussed Detector in 3D
- Modelling Sensor Directivity in 2D
- Modelling Sensor Directivity in 3D
- Focussed 2D Array with Directional Elements
- Filtering A Delta Function Input Signal

## See Also

`applyFilter`, `smooth`, `spectrum`
